# The Significance of Witness Sensors for Mass Casualty Incidents and Epidemic Outbreaks

**DOI:** 10.2196/jmir.8249

**Published:** 2018-02-02

**Authors:** Chih-Long Pan, Chih-Hao Lin, Yan-Ren Lin, Hsin-Yu Wen, Jet-Chau Wen

**Affiliations:** ^1^ Research Center for Soil & Water Resources and Natural Disaster Prevention National Yunlin University of Science & Technology Douliou Taiwan; ^2^ Department of Emergency Medicine, National Cheng Kung University Hospital College of Medicine National Cheng Kung University Tainan Taiwan; ^3^ Department of Emergency Medicine Changhua Christian Hospital Changhua Taiwan; ^4^ School of Medicine Kaohsiung Medical University Kaohsiung Taiwan; ^5^ School of Medicine Chung Shan Medical University Taichung Taiwan; ^6^ Department of Clinical Medicine West China School of Medicine Sichuan University Sichuan China; ^7^ Department and Graduate School of Safety and Environment Engineering National Yunlin University of Science & Technology Douliou Taiwan

**Keywords:** social media, mass casualty incident, internet, sensor

## Abstract

Due to the increasing number of natural and man-made disasters, mass casualty incidents occur more often than ever before. As a result, health care providers need to adapt in order to cope with the overwhelming patient surge. To ensure quality and safety in health care, accurate information in pandemic disease control, death reduction, and health quality promotion should be highlighted. However, obtaining precise information in real time is an enormous challenge to all researchers of the field. In this paper, innovative strategies are presented to develop a sound information network using the concept of “witness sensors.” To overcome the reliability and quality limitations of information obtained through social media, researchers must focus on developing solutions that secure the authenticity of social media messages, especially for matters related to health. To address this challenge, we introduce a novel concept based on the two elements of “witness” and “sensor.” Witness sensors can be key players designated to minimize limitations to quality of information and to distinguish fact from fiction during critical events. In order to enhance health communication practices and deliver valid information to end users, the education and management of witness sensors should be further investigated, especially for implementation during mass casualty incidents and epidemic outbreaks.

## Background

Due to the increasing number of natural and man-made disasters, mass casualty incidents occur more often than ever before. As a result, health care providers need to adapt in order to cope with the overwhelming patient surge [1]. To ensure quality and safety in health care, accurate information in pandemic disease control, death reduction, and health quality promotion is critical [2-4]. However, obtaining precise information in real time during a mass casualty incident is an enormous challenge to all researchers in the field. In this paper, innovative strategies are presented, in order to develop a sound information network using the concept of “witness sensors.”

## What Are Witness Sensors?

The term “witness” can be found in vital sectors of several medical fields, especially in emergency and disaster medicine. For instance, many reports emphasize the crucial role of bystanders in out-of-hospital cardiac arrests. These bystanders perform cardiopulmonary resuscitation or use automated external defibrillators [5-15]. When it comes to disaster medicine, the first witness usually initiates the systems of pre-hospital emergency medical services (EMS). However, while continuous messages from witnesses flood social media, a major dilemma arises. Should quantity of data be valued over quality? In order to overcome the reliability and quality limitations of the information emerging from social media, researchers must pay significant attention to developing solutions that verify the authenticity of the messages, especially for matters related to health. In this research, we introduce a novel concept that is based on the two elements of “witness” and “sensor” to address this challenge.

Witness sensors respond in a similar way to physical sensors, since they interact and report in real time. Physical sensors are calibrated and modulated prior to any operation so that only the signals from qualified physical sensors are accepted. Likewise, for the witness sensor, training, management, and accreditation policies are critical and should therefore be further investigated prior to executing this concept.

## Why Witness Sensors?

These days, the use of social media is an integral part of our daily life. As a consequence, information from witnesses can spread fast and far during catastrophic events [16]. This active and multisourced information is essential during disasters, and it leads to a huge number of messages and posts flooding the Internet. However, the quality and accuracy of some of these data may be questionable.

Nevertheless, the value of witnesses should not be ignored, especially in applied medical practices. For example, a magnitude 6.4 earthquake struck southern Taiwan at 03:57 on Feb. 6, 2016, and caused 513 injuries [17]. Remarkably at 04:05, several messages and images were reported in a discussion group on the widely used instant communication/social media app “LINE” in Taiwan, administered by local emergency medical technicians (EMTs). The major EMS responders were dispatched to the disaster area a few minutes later based on the details provided of the damage. Additionally, all the information was monitored by the fire department, response hospitals, and local government.

Owing to the high-quality information from the local EMTs, a temporary Emergency Response Center was set up immediately by the local government at 06:00 to manage and control the event. The incident command system responded efficiently, due to the precise information given primarily by specific witnesses.

However, this does not always apply in general witness cases. Two individuals shared fake posts on social media causing people to panic during a magnitude 6.0 earthquake that occurred in the same city a year later, at 01:12 on Feb. 11, 2017. 

**Figure 1:**
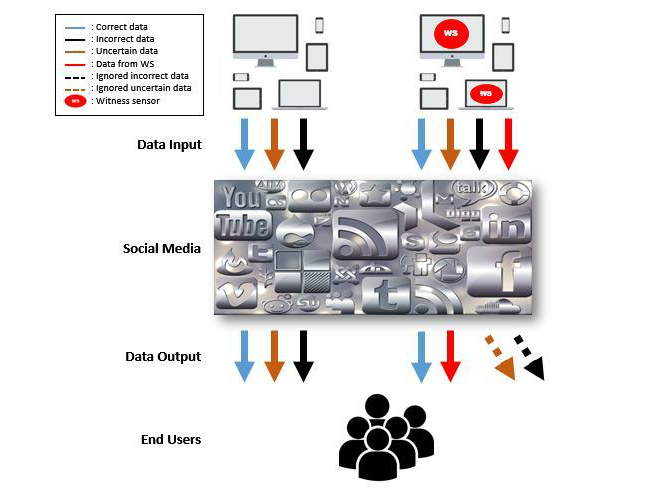
The different information pathways during a disaster between witness sensors and non-witness sensors.

Such phenomena appear globally and although there are mechanisms to prevent such incidents, retrieving and verifying information from witnesses is still a challenge to researchers. The concept of witness sensors is illustrated in Figure 1. Incorrect and questionable information will be ignored while the data from witness sensors are applied as principal references. In addition, qualified witness sensors could ensure the validity and dependability of communication, potentially leading to a paradigm shift in social media.

## Similar Cases of Witness Sensors

In Taiwan, several cases based on witnesses and/or volunteers have been applied successfully, mainly for the inspection of traffic and flood events. Real-time reporting of traffic conditions is one such case, which has been well implemented by the Police Broadcasting Service (National Police Agency, Ministry of Interior, Taiwan), online and on radio. The Police Broadcasting Service collects all the incoming information from witnesses and then disseminates that information to road users and to relevant agencies for adequate response and treatment. However, once again, the information quality from general witnesses is not always accurate and truthful, which leads to the need for additional confirmation and accreditation processes by other witnesses or traffic police officers.

This is one of the reasons why the witness sensors should be differentiated from general witnesses; qualification criteria are needed in order to be approved to provide valid information. The precise data collected from witness sensors could produce more reliable and valuable information for all users, especially during or after extreme events.

## Training Plan for Witness Sensors

The central dogma of witness sensors is to prevent or reduce the loss of lives during extreme events. The information given by qualified witness sensors could act as a reliable and safe source to corresponding decision makers, compared to data obtained from average social media users. The information pathway is similar to that of social networks, but the response behavior and purpose aim to direct accurate observations to emergency needs instead of personal opinions and embellishments.

Based on our strategy, volunteers will be invited and classified to three levels of witness sensor (WS): WS I, WS II, and WS III. The training goal of WS I is to report on-scene events with true facts, while WS II must report substantial evidence, as well as participate in measurement and evaluation practices. The WS III is a professional level of witness sensors, responsible for in-depth investigations and comprehensive interpretations. In this case, EMTs and members of Disaster Medical Assistance Teams would be the most appropriate candidates for WS III. Each level of witness sensor may not only act as an information peer, but also contribute to the emergency response of affected groups, when necessary.

## Benefits to Health Quality and Safety

Many researchers have welcomed the general benefits of social media to public health [18,19]. Some of the benefits include increased interaction with different groups, open source information, increased accessibility to global health information, support at multiple levels (eg, peer, social, emotional, as well as public health surveillance), and potential to enhance health policies. Even so, there are many limitations when using social media, especially in developing a robust and comprehensive evaluation mechanism to complement the quality concerns and lack of reliability [20].

Hence, the messages and details from witness sensors could not only promote quality information shared through social media, but also become a prototype model for screening the enormously uncertain health information that resides on the Internet. The witness sensors could improve the reliability of primary data and accelerate the tracking of information sources, since these are fundamental issues during pandemic outbreaks or catastrophic events.

### Conclusions

Social media and information exchanges on the Internet have become a major communication tool for health providers and users. In this paper, we proposed witness sensors as key players designated to alleviate possible limitations to accuracy of information and to distinguish fact from fiction during critical events. In order to enhance health communication practices and deliver valid information to end users, the education and management of witness sensors should be further investigated, especially for implementation during mass casualty incidents and epidemic outbreaks.

## Abbreviations

EMS: emergency medical service
EMT: emergency medical technician
WS I-III: witness sensor level I to III
